# The President’s Address

**Published:** 1889-08

**Authors:** W. W. Dawson

**Affiliations:** Cincinnati


### COMMUNICATIONS.

### The President's Address.

BY W. W. DAWSON, M.D., OF CINCINNATI.

Delivered at the fortieth annual meeting of the American Medical Association, Newport, R. I., June 25, 1889.

The Premier, Mr. Gladstone, after quoting the statistician who estimates the English-speaking people at the close of the next century at one thousand millions, says: " What a prospect is that of many millions of people, certainly among the most manful and energetic in the world, occupying one great continent." This destiny in numbers is startling, but the assertion of Dr. Dollinger, a German scholar, portrays the culture of the future almost as strikingly when he says " that the intellectual primacy of the whole world is certain to fall to the Anglo-Saxon race." Most of that race will be in America.

Looking to such a future the position of the learned professions is certainly conspicuous—their obligations imperious. Medical men should be loyal to this grand destiny.

An eminent modern critic, in discussing civilization in America, while admitting that we have well solved the political and social problems, asks what have we done to solve the human problem, " the humanization of man in society." The struggle in his own country, he asserts, has resulted in " an upper class materialized, a middle class vulgarized, a lower class brutalized."

We trust that our efforts have yielded better fruit; and since medical science and medical men are prominent factors in society, among every people, we may well ask, what have they accomplished, what part have they here taken in the solution of the vital problem? In the "*Century of Medicine*," Prof. E. W. Clark, in his classical address says:

"It is not an extravagant assertion to say, that in all this turmoil, change and progress (referring to the revolutions and changes in society, religion and government for the past century), medicine has kept abreast of the other natural sciences, of politics, and of theology, and has made equal conquest over authority, error, and tradition," and it may be added, has contributed largely to man's comfort, happiness, and advancement. To intensify this, reference need only be made to some of our triumphs, to vaccination, to anæsthesia, to sanitation, the prevention of pestilence, the lengthening of human life. It is, however, more especially the contributions of the profession in America to which attention is desired at this time. What are we doing in the humanization of man, in the work of civilization?

Are our medical practitioners and our medical teachers what they should be? We shall see. Criticisms abound concerning the defects of medical education. Those who do not condemn, often ridicule. These criticisms and strictures are made for the most part, it must be said, by gentlemen unacquainted with teaching, without any practical knowledge of the constitution of medical colleges, or of the toil, devotion, and sacrifice made necessary by those engaged in didactic and clinical instruction.

These censorious addressess are delivered before and to a body of professional gentlemen, the peers of any, some of whom have grown gray in the hard service, others are still in the prime of life, with reputations coextensive with civilization. The rest are young, full of life and enthusiasm, fired with ambition to render loyal service to that profession which they have chosen. Can our system be so defective? The pessimistic orator seems to forget that he is the product of the system of medical education which he is so severely condemning. Some one has said, "By retrospection and introspection an individual, like a profession, may be benefited." In this self-examination we should have but one motive, the elimination of error, the development and support of truth.

Education can not make all great or equal. It tends, however, to make all safe. In the crucible of private, practical life, evolution asserts itself and the fittest survive.

In making a retrospect of our profession it may be well to look for a moment at medical teaching in this country.

The way is long between Aristotle and Bichat, and Buckle says that he found no middle-man in this long period; it is darker than it is long. During all this time medicine was not taught legitimately. The renaissance, if it may be so called, began with Hunter and Bichat. No real progress, however, could be made while oxygen remained locked in the silent embrace of all organic and inorganic nature. Priestly, escaping from the religious and political contests, and it may be persecutions of the old world, came to this country to demonstrate his great phlogiston, oxygen.

Bichat and Hunter restored the proper study of medicine. They represent the turning-point from idealism, speculation, and theory, to accurate and close observation. The latter, John Hunter, in 1767, was lecturing and taking students into his own house, and it is curious to know that here, in far off America, Shippen and his contempararies in Philadelphia and New York, about the same time, or very soon after, began teaching medicine and surgery upon essentially the same plan. Of these men, one who so recently passed away that you can almost hear the sound of his voice and feel his magnetic presence, when speaking of the men who lived at the close of the last, and during the early part of this century, said, and justly said, "Not a few of them were the worthy peers of Roux, Abernethy, Crampton, Bell, Graefe, and Scarpa."

To quote again ;

"During the past century medicine has been enfranchised from superstition, *quasi*-charlatanism, bold empiricism and speculation, and has developed into a symmetrical science, affiliated with the other natural sciences, studied by the same methods and by the same appliances as they are, and like them, has been planted upon the solid basis of fact and demonstration."

It may be profitable for us to inquire and determine what part the profession in America has taken in placing medicine upon the high ground which it occupies. What have we done, what are we doing, and what forecast can we make of the future?

At the close of the eighteenth century, Boerhaave declared that all that had been learned up to that time was comprised in three propositions "Keep the head cool, the feet warm, the bowels open." All other pages in the volume which he left were blanks. Many pages, however, it will not be denied have been filled during the present century. What have been our contributions? Have they been such as to rank us with the acknowledged conservators of mankind?

In giving attention to this subject let us for a moment reflect upon the peculiar position of the profession and of medical teaching in this country. For many years (and even now) with few exceptions, medical colleges were the creation of the members of the profession, most often of the faculties composing the schools, without endowment—indeed, it may be said that almost every thing on this continent is endowed, except medical colleges—without governmental aid, depending for their support upon the sacrifice of time and money on the part of the gentlemen occupying the chairs. Yes, not only without patronage from the government, but society, from some unknown cause has ever been against legitimate medicine, depending upon the scientific physician in time of trouble, yet, in the interim openly supporting all sorts of shams, frauds, and impostors.

Elsewhere college work is provided for by the State; especially laboratory investigations—the nature and the genesis of disease. Hence it is not strange that in such departments we may not be so far advanced as our European brethren; but while they have been engaged in experimental studies, we have developed the practical. But everywhere is seen among us an earnest, a burning desire for higher culture, for more exact and accurate knowledge. Especially is this true of our younger members and of those about entering the profession.

A movement is being made to concentrate those who have had preliminary advantages—those who enter the profession as college-bred. No objection can be urged to this if it be not too exclusive. All efforts, in fact, to refine our profession without emasculating it should meet with judicious approval.

THE PHYSICIAN OF THE FUTURE.

Whence are medical students to come? What facilities are now afforded, and what does the future promise for the education of our young men, the class from which the medical student, the "coming doctor" is to be selected? The answer to this question will give some comfort, we trust, to the pessimist, and soothe the restless and at times unreasonable critic. And now as to our resources for this work.

By the last census it was shown that nearly four thousand institutions—schools for higher learning—existed in the United States, and that nearly four hundred of them ranked as colleges or universities. In these are massed yearly sixty thousand pupils. They, together with two hundred thousand common, or primary schools, in the higher grades of which the curriculum nears that of many colleges at home and abroad a third of a century ago, may be looked upon to supply year after year a better material from which medical students will be drafted.

Prof. Charles W. Eliot, in his beautiful and forcible centennial response, enumerated our educational facilities most generously. He painted our future more hopefully when he spoke of the 8,000,000 children in elementary schools, 250,000 in secondary schools, 60,000 in colleges, with 360,000 teachers to train and develop them.

Every one traveling through the states—especially those of the west and south, and those situated in the far away mountains, and on the Pacific—must be impressed with the onward march of public instruction, the gradually increasing general intelligence, and the vast sums that are annually expended for the education of the people. Public school buildings by their size, adaptation, and attractive surroundings, give an impression which the most skeptical must feel, a promise of the future which can not be misread. From such as these scientific medicine must reap a share. Every teacher, every one connected with the examination of candidates for the medical degree, knows—and the knowledge is reassuring—that year after year the grade of the medical student is advancing, that the material out of which the practitioner is made is constantly growing better, becoming stronger; in other words, that the preliminary education of our students is steadily becoming more broad and comprehensive. I gave utterance to this view a few years ago in an address which I had the honor of delivering to the State Medical Society of Ohio. Time I believe has confirmed what I then said. This confirmation is seen in our graduates as they go forth to take up the line and battle of life. Are they not the equals of the graduates in other professions, in law and theology? As life advances are they not the peers of any in all the useful elements of true manhood? Are they not the citizens of best rounded characters, citizens most relied upon by their neighbors in foul as well as fair weather?

Again, in addition to facilities already referred to, the most generous provisions are being made, all over our land, for institutions which will be worthy to be called Universities. From these, graduates will emerge worthy to rank by the side of those bearing the prized degrees from Oxford, Cambridge, Paris, Heidelberg or Leipsic.

During the summer of 1888, I witnessed the beginning of a University in California, which in scope and equipment will surpass, probably, any school upon the continent. Should Governor Stanford live to develop his conceptions, that far off State will have an institution of which not only the Pacific coast but our entire country, yes, all civilization, will feel justly proud. It may be so liberally endowed that it will command the best abilities of the world.

Of course, upon such an occasion as this, it would hardly be expected that I should in detail refer to the many liberal donations and bequests which have been made, by generous citizens, for developing higher culture—a more comprehensive education. I will, however, be pardoned for referring to a few, and I may say, without being too enthusiastic, that the future is aglow with promise. The high-hearted examples which have been set will be followed by other favorites of fortune, and this country may surpass the world, not only in common schools but in her institutions for broader and deeper education.

In looking at this promising future may we not hope that, before another half century closes, students from the old world will flock to this to sit at the feet of the wisdom here installed? Is it too much to hope that, in the not far off future, the preliminary education of our students will be equal to that required in the best schools of the world?

Defective as has been much of the material, yet have we not produced some marked results? Our best are equal to the best anywhere; mediocrity always and everywhere finds its own. The poor in medicine, the weak brother, however much we may deplore him, however much we may train him, we have, like the poor, always with us. This is the lot of humanity in all lands, among all peoples, new or old. A word as to the physical qualities of "the coming doctor." Recently a distinguished foreign traveler, in speaking of our educational facilities and national peculiarities, said: "Students are much calmer than their colleagues in Europe. They don't at all trouble themselves about politics or affairs outside their line of duty, and with the practical sense which animates the nation, they try to make the best use of their time. They fight no duels, and it is only for health and recreation that they take part in various sports and games." These remarks apply with equal, in fact, with greater force to medical students.

It is to the country schools, not to the city bred, that medicine must look for many of her strong recruits. Cities too often emasculate—young men are vitiated by indulgence and vice before they become possessed of serious thoughts, before they realize the elements of a healthy, vigorous life. It is this country-bred, this excellent material which is, as we have seen, yearly growing better and better qualified to enter upon the duties of the profession. From these we must look for the men of distinction, the leaders of the future.

Is this picture overdrawn? One word more. In many of the States of the Union, in addition to the liberally supported free schools and schools for higher education, already colleges have been established through the munificence of the General Government, in which the degrees of A.B. and A.M. may be obtained. They are absolutely free colleges, at which the poorest boy in the commonwealth may receive a classical education. And here you will allow me to say we can not insist too strongly upon the necessity of classical education; without it the medical man must ever be at a disadvantage. Without a knowledge of Latin and Greek sure and distinguished success is uncertain. The student may neglect Algebra and the Higher Mathematics, but let him, by all means, have a liberal knowledge of languages.

At the last commencement of one of our western schools, "40 per cent. of the graduating class had been admitted on diplomas from literary or scientific colleges. The balance of the class had received from one to five years of academic or collegiate instruction." This college is without endowment—depending entirely upon the learning, devotion and sacrifice of the faculty.

But to return. Prominent among the States in providing institutions for advanced culture, the great frontier State, Texas, claims a high position. The University of Texas will be one of the most liberally endowed; millions of acres of land have been donated for university purposes. There, in that Empire State, may yet be seen one of the greatest schools of literature, science and philosophy on the Western Continent. The University of Virginia, projected in the early days of the century by her great commoner, Thomas Jefferson, has yearly sent forth graduates equal in all the elements of advanced scholarship to those from any school. This may seem high praise, but the records of her alumni justify me. The same may be said of Harvard, Yale, Princeton, Columbia, Cornell, and most of our older institutions. The University of California has already an annual revenue of about $200,000. The University of Michigan, with a yearly income of almost a quarter of a million of dollars, has well nigh two thousand students, taught by more than one hundred teachers.

Let us not, gentlemen, be impatient; the influences are already projected which will give us students equal to—up to—the highest standard of preliminary preparation. If we have accomplished so much in our primitive stage, what may we not expect when all our great preparatory works come fully into action?

From this view of the resources from which medical students are to be drawn, and of the liberal preparations and facilities for their culture, we may well ask, what is the profession doing to profit from such advantages?

Some of the classical schools at Oxford and Cambridge were organized as early as the thirteenth century, but the systematic, scientific study of medicine and surgery came long subsequently —not for four hundred years later—about the middle of the eighteenth century. It was first projected in Great Britain, and soon after in our Atlantic cities. Unlike the Old World, our fathers had a wilderness to conquer before progress could be made. When the Pilgrim Fathers left England, reading and writing were rare accomplishments; chimneys in that country had just been invented and flock beds were luxuries. The adventurers—the emigrants to these shores from that ancient and imperfect civilization—had much to learn, but in the midst of their pitiable ignorance, facing great hardness and pressing wants, they were quick to provide educational opportunities for all. The result of their efforts are apparent—they are before us. Could more have been accomplished in one century?

MEDICAL SCHOOLS.

Our medical colleges now number a few more than one hundred. They may be classed as: 1, Metropolitan, those in large cities. 2, Medical colleges in less pretentious cities. 3, Medical colleges in small cities, 4, State medical colleges. For convenience, however, we may speak of them as Metropolitan and Provincial.

Before speaking more definitely of our medical institutions, allow me to refer, for a moment, to the proposition that medical schools in our country have been developed by the labors, and the self-sacrifice of the profession. As previously stated, it may be said that in this country everything is endowed except medical colleges, schools for teaching medicine. Yes, all financial responsibilities have been and are assumed by the faculties, by men who give every hour not devoted to "earning the guinea" to college work, and in most instances without pecuniary reward. It is only recently that the wise, the generous, the favorites of fortune, and a few of the States, have conceived the idea of endowing medical schools, institutions where medicine and surgery can be cultivated without the embarassments of financial responsibility. In the presence of such facts, the work of the grumbler seems indeed ungracious.

In our Metropolitan colleges every physician may feel a just pride; their graduates, most of them, will compare favorably with those educated anywhere on this earth.

The accomplished Dr. Senn, after a liberal experience with foreign schools, said: "There is no question in my mind that the average American student learns more in one month than the average German student in three. He learns more, not because he has better teachers or better facilities, but he makes better use of his time. I am satisfied that in our last graduating class I had at least a dozen students who, after studying three years, would pass a brilliant examination in any English or German university. They would have felt at home even in a dress coat in Volkmann's Klinick, passing their final examination."

Provincial schools do praiseworthy, yes, thorough work in training young men, not only in rudimentary branches but in practical, clinical studies. Many supplement these by hospital attendance in the great cities and by post-graduate courses. It is gratifying to know that these organizations are being established in all of the great medical centres.

The advance in medical education is again most distinctly pronounced by a remark recently made by one of our distinguished fellows, an American-bred physician, of whose fame we are all justly proud. In a conversation Dr. Batty said: "When I began the practice, thirty years ago, there was scarcely a graduate within fifty miles of my residence; now, however, there is hardly a practitioner in the same territory who is not a graduate, and, year after year, a portion of our young men leave home to avail themselves of clinical advantages, to attend post-graduate instruction." Could anything show more forcibly the conservative and steady growth of medical culture?

HAVE MEDICAL COLLEGES INCREASED TOO RAPIDLY.

Should they be established in small cities where clinical material is limited, where it must be comparatively scarce?

Before answering this, it may be well to reflect upon the proposition, that in our own country, as well as elsewhere, great achievements have often been made in the Provinces and not always under the shadow of the Universities. One of the great operations waited for years for a metropolitan disciple—one to take it up—and that too, long after the provinces, at home and abroad, had demonstrated its vital utility, its claim upon the scientific and skillful surgeon.

As our population increased from three to sixty-five millions, the demands for medical men were great—colleges increased necessarily. Have they multiplied in undue proportion?

In answering this question, I beg again to quote from my beloved master, Samuel D. Gross, to whom this question had been put. After mature deliberation, he said: "Our colleges are not annually graduating one physician for each county in the States and Territories. This is certainly not exceeding the demand." A considerable proportion of those who graduate never enter the ranks—death and desertion claim a large share. It would simply be impossible for the metropolitan schools to graduate all required.

For the introduction of young gentlemen into the profession, there is a mutual responsibility between teachers and perceptors. In very truth it may be said that colleges do their duty, their very best, with the students furnished by the preceptors. Give us liberally educated young gentlemen, and we will furnish graduates worthy of the degree. Medical colleges, however, do not make the physician. They merely furnish the foundation work; the individual must do the balance. In no place is evolution so marked—the fittest will and should survive.

### LABORATORY WORK.

Huxley says: "The microscope extends the realm of Pathological Anatomy to the limits of the invisible world."

"The intimate alliance between morphology and medicine has made the natural history of disease attain a remarkable degree of perfection."

Dr. George M. Sternberg, the distinguished pathologist, recently connected with the Smithsonian Institution, in referring to some of the laboratories established in this country for the study of pathogenic micro-organisms, says: "It is no longer necessary to go abroad for instruction in this department of science, since the laboratory of Prof. Welsh, in Baltimore, and the Hoagland Laboratory, in Brooklyn, afford facilities which are unsurpassed by any of the laboratories of the old world."

Indeed, it may be said that provisions for the study of pathogenic micro-organisms are established in most of the leading schools of this country—in New York, Philadelphia, Boston, Baltimore and the cities of the west and south.

You will pardon me for mentioning some of the investigators. Johns Hopkins' University has for its director, Prof. William Welsh. The Hoagland Laboratory, of Brooklyn, New York, established through the generosity of Dr. C. N. Hoagland, has been built and equipped in the most complete manner for research work in bacteriology and experimental pathology. Prof. George M. Sternberg is to be the director of this advanced institution.

At the University of South Carolina, Dr. Meade Bolton, who has had the best of training at Berlin and Gottingen, is at the head of a laboratory. Dr. H. C. Ernst has the direction of a bacteriological laboratory in connection with the Harvard School of Medicine.

Prof. James T. Whitaker, who had the honor of being the first American student of Robert Koch, demonstrated, at Cincinnati, in 1882, the tubercle bacillus, after a lecture upon the subject before the Philadelphia Academy of Medicine. In 1887 the Medical College of Ohio imported a complete outfit for bacteriologic study. The conductors of the laboratory, Drs. Rachford, Cameron and Freeman, during the first course, had the opportunity of doing some good work in the discovery of the typhoid bacilli in the reservoir supplied from the Ohio river then at a very low stage. The discovery led to the general adoption in the city, as advised by leading physicians, of boiling all drinking water, a plan which undoubtedly limited the spread of the disease.

Among others may be mentioned Dr. Prudden, of the College of Physicians and Surgeons, of New York; Dr. Geo. A. Kemp, of Brooklyn; Dr. Mall, of Baltimore; Dr. Booker, of the same city; and Dr. Frank S. Billings, of Lincoln, Nebraska.

One of the earliest, most accomplished and acurate cultivators of micro-organisms, is Dr. James E. Reeves, of Chattanooga. His technique is singularly beautiful. Many of his preparations are to be found in the National Museum.

Dr. Victor C. Vaughan and Dr. Heneage Gibbs conduct laboratory work at Ann Arbor.

The University of Pennsylvania has at the head of its laboratory Dr. John Guiteras, a pathologist who has distinguished himself in the study of the origin and spread of yellow fever.

Thus it will be seen that in all parts of our country—East, West, North and South—laboratories are being established for original work.

### HISTORY OF MEDICAL TEACHING IN THIS COUNTRY.

A brief review of medical teaching in this country will be pardoned—it may be profitable—it will certainly illume the present, and may be somewhat of interest to the future.

The first medical lectures were delivered by Dr. John Morgan and William Shippen, in 1767, in Philadelphia. Dr. Rush and Dr. Physic soon after participated, and in 1768 the medical department of the University of Pennsylvania was organized; that great school which is steadily advancing to the highest station. Philadelphia was a small, a provincial city at that time; now she is only second to the great metropolis in numerical strength, but second to none in the thorough equipment of her medical schools.

Contemporaneous with Philadelphia, an organization was projected for medical instruction in New York. In 1767, the first steps were taken which resulted in the school, ever since known as the "College of Physicians and Surgeons," one which challenges the confidence of all. The medical colleges of New York, endowed, not by government, but by her public spirited citizens, have won the honors which they wear so well.

In 1785 the first school was organized in Boston. The chairs were four, and the session four months. Harvard is the outgrowth of this humble beginning of that provincial faculty.

In 1800, the first medical instruction was given in Baltimore; since then, the schools of Maryland have occupied a deservedly high position. Recently one of her citizens made an endowment by which the "Johns Hopkins University" will be equipped for the most thorough work, experimental work, laboratory studies, a range and grade of investigations *en report* with the spirit of the times.

This great benefactor has also given to Baltimore one of the most completely equipped hospitals to be found on this earth.

The great Mississippi Valley was yet unknown, but soon after the close of the Revolution, emigration began, and as early as 1799, Dr. Samuel Brown organized the medical department of Transylvania University. Dr. Benjamin Dudley effected a reorganization in 1819. This school, after many prosperous years, having graduated men who acquired distinction at home and abroad was transferred, or rather, most of the faculty removed to Louisville, when and where the University of Louisville was founded.

During the early part of the century, medical schools were organized in several of the eastern states, usually under state or church patronage. Most of them exist to-day. Some of the most distinguished men in our profession have been associated with these institutions.

As the west and south were peopled, medical schools were established in cities and promising towns. As early as 1819, Dr. Daniel Drake secured the charter of the Medical College of Ohio, and had it legally connected with the City Hospital. The faculty constituted the hospital staff, the members of which were required to give clinical lectures—the first forward step on the continent, in blending didactic with clinical instruction.

The physicians in South Carolina began medical teaching in 1823, and those of Louisiana in 1835. In both of these states schools of high character have been maintained.

In closing this very brief review of our colleges, Metropolitan and Provincial, I think it may be said that year after year the standard of the doctorate is being elevated, preliminary examinations and graded courses are being adopted, the smaller schools, to which most blame is attached, whether justly or not, with a disregard of self-interest seldom seen, are yearly reducing the size of their classes by insisting upon higher preliminary education, by extending the curriculum and by graded instruction.

MEDICAL JOURNALS.

Medical journals, Metropolitan and Provincial, are the heralds, the vanguards of medical progress, the exponents of professional culture. They are closely associated with the colleges in education and in post graduate instruction. In them appear the best thoughts of the best men; they constitute the great forum of intellectual combat; upon their pages pretension is analyzed and estimated, and worth recognized; that which is new or original is endorsed, or rather encouraged; it is only the plan, the original investigation which is endorsed; the results, the conclusion must be subject to the crucible of test and trial.

The London *Lancet* and the *American Journal of the Medical Sciences* were almost contemporaries—who can overestimate their value—their influence in medical progress. While our journals, both Metropolitan and Provincial, are freighted with the best thoughts of the best men, yet, it must be confessed, that trash and light material—very light material—may be found in all, but the reader, nevertheless, will find much that is not worthless.

The *Journal* of this Association has won its way to its present high position by its dignified course and its essentially scientific character; but has it reached its full usefulness? A learned and distinguished author, and a highly prized fellow of our association, at my suggestion, gives his views upon this question.

Dr. Comegys says: "The undertaking, seven years ago, to establish a weekly journal, was a happy conception, and has been carried on as successfully as the resources of the association would admit. To Dr. N. S. Davis unstinted praise is due; proportionate praise is also due the Board of Directors with whom he has been associated.

"A large number of the members believe that it is entirely feasible to enlarge The Journal and give to it increased capacity for usefulness; indeed, that it should be made more fully capable, as the organ of the Association, to assert and maintain the dignity and power of the medical profession as one of the greatest factors in civil life; that to it society must ultimately turn to find not only the resource to assuage the distress arising from the diseases and accidents of life, but as to its protection from all those evils that fill the land with apprehension of desolation and ruin.

"We know to what an immense extent we can estop the approach of the pestilences that desolate lands and which menace, through the paths of commerce, the whole area of civilization. We know what we can do to improve the homes and places of labor of the lower and toiling classes of cities and other crowded centres of population. There is nothing, indeed, connected with our own social state which the medical profession should not supervise and which it should not have the power to control. A great organ is necessary to enlighten, strengthen and lead the profession in all directions to bring to bear its benificent agency for the correction of the terrible evils of society.

"Such a journal must be made encyclopædiac in character, in which can be found the proceedings of distinguished societies of this country and of Europe, the work of the chief actors of medical progress in all parts of the world. Twelve thousand subscribers would give $60,000, this would insure $40,000 from advertisements, making an income of $100,000, which would sustain one of the grandest journals in the world.

"The *British Medical Journal* in fifteen years has 14,000 subscribers and an income of $125,000. May we not hope to reach this, and when we do who can compass the good which the American Medical Association will accomplish?"

MEDICAL SOCIETIES.

Our medical societies, local and National, are great factors in professional progress. Not alone are they valuable for their social opportunities, but in and through them a vast amount of valuable matter is presented.

MEDICAL LITERATURE.

Had Sidney Smith been a physician and given to reading, he would not, even in 1850, have asked the questions: "Who reads an American book?" "What does the world owe to American physicians and surgeons?"

This reverend gentleman, this famous critic, could not have heard of Ephraim McDowell, whose brief paper, detailing his first three cases of ovariotomy, published in "The Philadelphia Repertory" in 1817, was of more value, did more for the conservation of human life than a score of ordinary publications. Our first half-century may be poor in books, but it abounded in strong, brave, conscientious and devoted men, men who, with the most limited resources, accomplished the grandest results. They compelled success because they deserved it.

The ink was hardly dry upon that cynical pen when anæsthesia was presented by the profession so poor, as he supposed, in valuable works.

But what country or age can match, in great contributions to the relief of the suffering, McDowell, Sims, Bigelow, Sayre, Batty and Emmett, and that trinity of men—Wells, Morton and Jackson—who gave anesthæsia to the world? Think of anæsthesia and of its influence upon the progress of medicine and surgery. But yesterday a writer in the London *Lancet* gave a graphic history of its reception in London; how the great Liston, having a patient who could not nerve himself up to the point of consenting to have a limb amputated for strumous disease of the knee joint, decided that "if the insensibility could be insured and maintained for one minute he would amputate." Reflect, for a moment, on the hesitancy of the great surgeon of University College Hospital, as he stood by the side of that patient; he could hardly believe the novel report as it came over the sea. Willing and anxious as he was to operate, he hesitated to urge the poor patient to make the experiment—experiment it then was. In a week, however, it was legitimate practice all over the world.

The heart of every American physician is filled with thankfulness when he remembers that in the Providence of God this great boon to humanity was vouchsafed to his country. The very ground upon which stands Massachusetts Hospital is sacred to us all. Associated with the discoverers must ever be the name of Dr. Hayward, who performed the first operation under the strange letheon. Previous to this, operative surgery was slow, tedious and almost cruel. Contrast it to-day with what it was previous to 1847. What grand strides it has made under the direct support of anæsthesia and its almost equal co-laborer, antisepsis; the great cavities are invaded and invaded safely; the abdomen has become a familiar field, and who can forecast the surgery of the brain?

Since Emmett's operation we hear no more, neither in this country nor abroad—neither in London nor Berlin, neither in Paris nor Vienna—of that culmination, that *ultima thule* of ignorance, "ulceration of the os." What a disgrace that term was to the surgery of the world!

The ignorance in diagnosis was only surpassed by the cruel treatment which it evoked, the application of caustics to the tender everted membrane of the cervical canal.

Has the operation, Bigelow's litholapaxy, the crushing and evacuation of a stone at one sitting, been truly estimated? Its adoption in one celebrated case might have changed the destinies of Europe. Previous to Bigelow, lithotrity was an uncertain, and, in most hands, a cruel operation: "Crush all possible at a short sitting, and allow the fragments to pass *via naturalis.*" Bigelow realized that if anæsthesia is safe for two minutes it is safe for two hours or more; hence, he said: "Crush it at once, and evacuate the bladder by an aspirator." The operation, in proper cases, is as practical as the description is brief and efficient.

The accomplished Edmund Owen, M.B., F.R.C.S., upon calculus, says: "With rare exceptions only two operations are now practiced—suprapubic lithotomy and crushing, with evacution at a single sitting." A high compliment from an eminent authority.

The story of Ephraim McDowell, though so often repeated, humanity never tires of hearing. To us he belongs, and to us only; we can not share his fame with another; we would not if we could. Who can measure the relief which his operation has bestowed upon suffering woman?—not only woman, for his was the genius which opened the way for laparotomy in both sexes.

### CLOUDS.

What has been accomplished by the profession in this country, self-reliant, and as we have heretofore said, without governmental or social support, is certainly worthy of congratulation, and gives ground for hopes of a rosy-hued future; but, alas! there are some dark clouds to be seen—some spots on our sun of promise! Have we inherent defects in our organic law—our *esprit de corps?*

Upon the face of our promising future some omens of evil appear, indications which look not up but down, not forward but backward, not to the elevation but rather to the degredation of our profession. Heretofore we were an organization into which no species of fraud could enter; pretension, ignorant pretension, stopped at the door. No *ism* or *pathy* was admitted; something more than a diploma, "a legal diploma," was required—a clean bill of conduct, free from false assumption, assumption of universal knowledge, of specific remedies, of imaginary potencies; in fact, of all shams and false claims, a guild in which there was the greatest freedom for the truth, the largest liberty for the right. No vendor of secret remedies was admitted, because of the ignorant presumption in which they were conceived and propagated; but, alas, that we should have fallen upon the evil times when "patented processes" are attempted, when "processes" of valuable remedies are kept secret. These remedies with "patent processes" are in daily use. This is one of the dark spots in the picture. It came in with the "legally qualified practitioner." What is antipyrin, antifebrin, salol, sulfonal? The reliant patient may well propound such questions. Who can answer them? Are we relegated at one fell move back into outer darkness, the associates of vendors of "secret remedies," of "patented processes?" What higher is a "patented process" than a "patented nostrum? The profession was never so low as to countenance the latter; but have we not, in these latter days, become propagandists of patented, and, therefore, secret processes?

### LAWS FOR THE REGULATION OF THE PRACTICE OF MEDICINE.

It may be asked, has the standard of professional excellence been raised by laws enacted in many of the States for the regulation of the practice of medicine?

These laws banish the poor creatures without diplomas, but make respectable, *quasi*-respectable, all who have so-called diplomas from whatever source. Shams and pretenders are in this way made "legal," and claim whatever protection and recognition that term may give or imply. A chartered institution, in most of the States, represents a formal application for incorporation to a Secretary of State, the signature of that officer, and nothing more. The process of graduating from such—the faculty often consisting of but a single person, or a man and his wife—would hardly be called a farce, the subject is too serious. "Legally qualified!" Think of it; and yet this legally qualified creature will claim and expect to meet the highest and the purest. Is this an advance upon the requirements of the Code, the morals and *esprit de corps* of which we have never been questioned?

What has been the effect of these diplomas? Let any candid man answer. Have they not tended to make vice and presumptious ignorance respectable?

Let us be true to ourselves; pitch can not be touch without defilement. Our profession must keep pure or else it will degenerate and sink to the level of a trade.

In State boards of health by the side of physicians we find these "legally qualified" practitioners. Where lies the responsibility? Is it with us? Our self-examination on this subject should be searching. If we have failed in our duties to humanity let us be swift to acknowledge it, and still more eager to correct our error.

The presence of this body of professional gentlemen, representing our entire country, furnishes sufficient argument for the existence of a national organization; one embracing the virtue and strength of the profession, one to which all questions should be referred for just and final decision. Questions will arise, differences of opinion will occur between honest men. We must have some tribunal, some body, to which these questions, these differences of opinion can be relegated for solution. The golden rule is a principle, not a law; it cannot interpret itself. Its application to life in detail must be defined. In this respect we are like other men and other organizations. Our *morale*, however, is higher; it has a zeal, a spirit, a hope and confidence peculiarly our own. If we would have our organization pure, we should make it strong--strong enough to eliminate all that is not true or truthful. We are mortals not transcendentalists. We can not live as the commune. We must have laws; remembering always that they are not made for the righteous, but for the sinner. "They that be whole need not a physician, but they that are sick." I would not attempt to defend the ethics of our profession. It will be a poor compliment to your intelligence, to your manhood; for there is not a clause in our code which a gentleman could not cheerfully obey. Organize whatever we may please: associations of specialists of physicians, of surgeons, academies of physicians, congresses of physicians and surgeons; but let us not lose our loyalty to this parent association. Projected almost half a century ago, when medical societies were few, it has annually convened—in the north, in the south, in the east, in the west, and in the far west on the Pacific shore. If you will examine its yearly roster you will find that it embraced the best and the wisest. Almost all who were present at the beginning are at rest; their places have been filled by worthy men. Thus yearly new life—new men being added--this association can not grow old.

"When a people hold their lives and property as nothing the enemy has already suffered defeat." So too when virtue will not compromise with vice, the victory, although it may be long delayed will surely come.

Of the American Medical Association let us unite in saying, *esto perpetua.*

---

An Incorrect Diagnosis.—A business man and financier of the first rank in Boston is so absent-minded that he occasionally forgets to go to his dinner. His customary hour for this meal—when he remembers it—is two o'clock. The other day, quite absorbed in business, he worked steadily on until four o'clock, and then began to have a quite natural sense of emptiness and yearning in his stomach. "Dear me," he said musingly, applying the flat of his hand to his waistcoat, "I wonder what I ate for dinner that disagrees with me?"—*Boston Transcript.*

